# “Unmasking Misinformation”: Characterizing the Social Media Landscape of Disorders of Consciousness on X (formerly Twitter)

**DOI:** 10.21203/rs.3.rs-10809019/v1

**Published:** 2026-09-16

**Authors:** Wasim Ahmed, Mariann Hardey, Vishakha M. Rao, Sara J. Stern-Nezer, Joseph Kass, Sarah Wahlster, Krista Lim-Hing, Neha S. Dangayach, Aarti Sarwal, the Curing Coma Campaign and its Contributing Members

**Affiliations:** 1 Hull University Business School, University of Hull; 2 Professor, Accessible and Equitable Technology, Durham University Business School; Advanced Research Computing (ARC), Durham University, Durham, United Kingdom; 3 The Awty International School, Houston, Texas, USA; 4 Department of Neurology, UCI School of Medicine, Irvine, California, USA; 5 Departments of Neurology, Psychiatry & Medical Ethics, Baylor College of Medicine, Houston, Texas, USA; 6 Departments of Neurology, Neurosurgery & Anesthesiology, University of Washington, Seattle, Washington, USA; 7 Department of Neurosurgery, Northwell Health, New Hyde Park, New York, USA; 8 Departments of Neurology and Neurosurgery, Icahn School of Medicine at Mount Sinai; 9 Department of Neurology, Virginia Commonwealth University School of Medicine, Richmond, Virginia, USA

**Keywords:** disorders of consciousness, coma, brain death, vegetative state, social media, misinformation, topic modeling, health communication, X (Twitter), neurocritical care

## Abstract

**Background/Objective:**

To characterize the discursive landscape of public discussion related to disorders of consciousness (DoC) on the X social media platform, including its nature, prevalence, thematic structure, and contextual diversity.

**Methods:**

Point-prevalence, observational study of social media content using a combined social network analysis and unsupervised topic-modeling approach. The setting was X (formerly Twitter), a global public microblogging platform. English-language posts published between January 1 and December 31, 2023 were retrieved using a curated set of nine DoC-related keyword phrases (“Disorders of Consciousness,” “Coma Recovery,” “Vegetative State,” “Minimally Conscious State,” “Brain Death,” “Locked-In Syndrome,” “Medically Induced Coma,” “Coma Prognosis,” “Dead Zone”). No interventions were applied.

**Results:**

A total of 30,621 posts were retrieved. Social network analysis using the Harel-Koren Fast Multiscale layout and Clauset-Newman-Moore modularity-based clustering identified seven principal user communities. BERTopic transformer-based topic modeling yielded 50 coherent topics, which were grouped through structured thematic synthesis into three dominant discourse domains: (1) medical and clinical (vegetative state, brain death, locked-in syndrome, medically induced coma, and prominent named cases); (2) ethical, legal, and societal (organ donation/procurement, end-of-life and beginning-of-life debates, criminal justice cases involving vegetative outcomes); and (3) figurative and non-medical (the term “dead zone” used in technology, gaming, popular culture, and Stephen King’s novel). Non-medical uses constituted a substantial fraction of total retrieval volume, and the unsupervised model was unable to algorithmically separate clinical from figurative uses of overlapping terms.

**Conclusions:**

Public discourse about DoC on X is substantial in volume, thematically heterogeneous, and embedded within emotionally charged, legally complex, and figurative contexts that dilute clinical meaning and create fertile conditions for misinformation. These findings provide an evidence-based map of where targeted educational and public-health interventions are most needed and underscore the value of cross-platform, multilingual, and domain-specific NLP follow-up work.

## INTRODUCTION

Social media has become a primary venue for the exchange of health information among the general public,^1^ and the rapid, low-cost diffusion of accurate health content can confer measurable population-health benefits. Curated dissemination of peer-reviewed scientific findings through social media platforms is associated with increased downstream readership of the underlying primary literature,^2^and platform-based health campaigns have been deployed at scale across multiple disease areas, including cancer prevention,^3–5^cardiovascular and behavioral health.^6^ The same characteristics that make these platforms efficient vehicles for accurate information, speed, scale, low friction, algorithmic amplification, also enable the propagation of inaccurate, biased, or sensationalized content with potential public-health consequences.

Disorders of consciousness (DoC) are an umbrella of clinical states that follow severe acquired brain injury, including coma, unresponsive wakefulness syndrome (formerly the vegetative state), the minimally conscious state, and post-traumatic confusional state.^7^ Substantial international heterogeneity exists in how DoC are clinically defined, evaluated, and prognosticated, with the recent Curing Coma Campaign international survey documenting wide variability in operational coma criteria, etiologic mix, and use of advanced neurodiagnostics across 41 countries.^8^ This clinical complexity, layered on top of profound prognostic uncertainty, makes DoC particularly susceptible to misinformation, both within the healthcare system and across public discourse. Misinformation can in turn skew family expectations and clinical decision-making, contributing either to therapeutic nihilism that prematurely limits care^9^ or, conversely, to inflated hope for recovery in patients who will not meaningfully regain consciousness. Persistent gaps in family- and clinician-facing educational resources have been recognized as a priority area by the Curing Coma Campaign.^10^

Despite the recognized importance of public engagement and education for DoC,^9^ the existing empirical literature characterizing how the public actually discusses these conditions on social media is sparse and largely confined to single-language or single-platform analyses. Recent work analyzing Japanese-language YouTube and X content found a high prevalence of non-informative or actively misinformative “brain death” content driven in part by political and pseudoscientific actors,^11^ and a crowdsourced survey of lay perceptions of the vegetative state/unresponsive wakefulness syndrome identified marked confusion regarding the relationship between behavioral unresponsiveness, awareness, and prognosis.^12^ To our knowledge, no prior study has systematically characterized the broader English-language X discourse spanning the full DoC taxonomy.

In this study we describe the social media landscape surrounding DoC by combining social network analysis and transformer-based topic modeling of one calendar year of X posts retrieved using a curated DoC keyword set. Following recent guidance for reporting Twitter-based health research,^13^ we explicitly frame this work as a discourse-mapping study: our objective is to characterize the contexts and communities within which DoC information, accurate and inaccurate, circulates, rather than to directly adjudicate post-level accuracy. The resulting evidence base is intended to inform the design of targeted public-facing education, advocacy, and counter-misinformation efforts by clinicians, patient organizations, and neurocritical care societies.

## METHODS

### Study Design and Reporting

We conducted a point-prevalence observational study of public X posts. Reporting follows applicable elements of the Sinnenberg et al. checklist for Twitter-based health research^13^ and the STROBE statement for observational studies adapted to the social-media context.

### Data Source and Retrieval

We retrieved English-language posts published on X (formerly Twitter) between January 1, 2023 and December 31, 2023. Data extraction was performed on November 4, 2024 using NodeXL Pro, a social network analysis package implemented as a Microsoft Excel add-in. Retrieval used the Twitter Academic Track API (as it was known then) and post-level data included originals, replies, quote posts, and reposts; the cohort included all interaction types other than “likes.” Calendar year 2023 was selected as the analytic window because it post-dates the elevated COVID-19-related social media activity of 2020–2022 and predates the substantial United States socio-political activity of 2024–2025; this window therefore represents the most stable contemporary period for which a full calendar year of data is available. We acknowledge that retrieval ~10 months after the end of the analytic window may have under-sampled posts subsequently deleted, made private, or removed via platform content moderation; the magnitude of this attrition cannot be quantified retrospectively and is discussed under Limitations.

The keyword set comprised nine DoC-related phrases: *“Disorders of Consciousness” OR “Coma Recovery” OR “Vegetative State” OR “Minimally Conscious State” OR “Brain Death” OR “Locked-In Syndrome” OR “Medically Induced Coma” OR “Coma Prognosis” OR “Dead Zone.”* Hashtag mentions of these terms and replies to posts containing them were included. We deliberately excluded broad single-word terms (e.g., “coma” alone, “unconscious”) to limit retrieval of incidental, non-DoC uses such as “food coma” or “coma-inducing meeting.” The keyword glossary was developed by exploratory review of X posts surfaced by an initial search on “disorders of consciousness” performed September 9, 2024 and reviewed by three authors (A.S., M.H., W.A.). The inclusion of “Dead Zone” was retained from the initial glossary because, in our exploratory review, it surfaced legitimate clinical conversations about prognostically futile post-arrest states; we note in Limitations that this term also indexes a substantial volume of non-medical content.

#### Sample characterization:

We report the following descriptors of the retrieved corpus in the Results: total posts after the English-language filter; unique authors; ratio of original posts to reposts/quotes/replies; geographic distribution where inferable from user metadata; temporal distribution across calendar 2023; and median post-level engagement (likes, reposts).

### Social Network Analysis

User-to-user interaction networks (reply, quote, repost) were constructed in NodeXL Pro. Network visualization used the Harel-Koren Fast Multiscale layout algorithm^14^ and community detection used the Clauset-Newman-Moore modularity-optimization algorithm.^15^ Resulting communities were classified using the six-type Twitter network typology of Himelboim et al (2017). ^16^

### Topic Modeling

Topic modeling was performed using BERTopic,^17^ a transformer-based topic-modeling framework. Analyses were implemented in Python 3.x within the PyCharm IDE. Pre-processing was applied to English-language posts (identified using langdetect) and included URL, mention, and hashtag removal, removal of non-alphanumeric characters, lowercase normalization, tokenization, and removal of English stopwords using the Natural Language Toolkit (NLTK);^18^ non-English posts were retained in unprocessed form. Sentence embeddings were generated with the default all-MiniLM-L6-v2 model from the sentence-transformers library.^19^ Dimensionality reduction used UMAP^20^ and density-based clustering used HDBSCAN,^21^ both with BERTopic default settings (UMAP: n_neighbors=15, n_components=5, min_dist=0.0, metric=“cosine”). BERTopic was configured with min_topic_size=5, which sets the effective HDBSCAN minimum cluster size; nr_topics was left unset (no topic-count reduction was forced), yielding 50 topics. All topics and their top representative terms were inspected manually by the analytic team for face-validity coherence.

### Thematic Synthesis

After BERTopic produced its final topic set, the full authorship panel grouped topics into higher-order discourse themes by consensus through a series of virtual meetings, using a deductive-then-inductive coding framework anchored on the a priori distinction between substantive DoC discussion and incidental or figurative DoC term-use. Theme assignments were not coded independently; rather, consensus was reached collaboratively across the full panel, and disagreements were resolved through structured discussion during the virtual meetings until unanimous agreement was achieved. The final theme set, presented in the Results, consists of three discourse domains: medical/clinical; ethical/legal/societal; and figurative/non-medical.

### Misinformation Framing

Consistent with established definitions, we use the term *misinformation* to denote false, misleading, or context-stripped health information disseminated without regard to intent.^11^ We did not perform post-level adjudication of factual accuracy against a clinical reference standard. Accordingly, throughout this manuscript we report the *contextual landscape* within which DoC misinformation can circulate, not direct prevalence estimates of inaccurate content.

### Ethics

This analysis used exclusively publicly available, aggregated post-level metadata. No individual users are identified, and no posts are quoted verbatim. IRB review was not required for this study. All data handling complied with the X Developer Agreement and Policy in force during the retrieval window.

### Use of Artificial Intelligence

Claude Opus 4.8 (Anthropic) was used solely for copy-editing assistance on the manuscript draft. It was not used to generate, analyze, or interpret data, or to produce scientific content. All analyses, interpretations, and conclusions are the authors’ own, and all AI-assisted text was reviewed and verified by the authors.

## RESULTS

### Corpus Characteristics

The DoC keyword query retrieved a total of **30,621 posts** published between January 1 and December 31, 2023. The network comprised 27,054 unique users, though this figure reflects all vertices in the network—including accounts that were only mentioned, replied to, retweeted, or quoted, rather than distinct posting authors alone. Based on NodeXL edge-type counts, which tally relationships rather than discrete posts (a single tweet may generate multiple edges), replies predominated (59.0%), followed by originals (22.1%), reposts (10.4%), and quote posts (8.5%). Geographic metadata, posting-volume distribution, and per-post engagement were not available from the NodeXL output and could not be characterised.

### Social Network Structure

**Figure 1.**
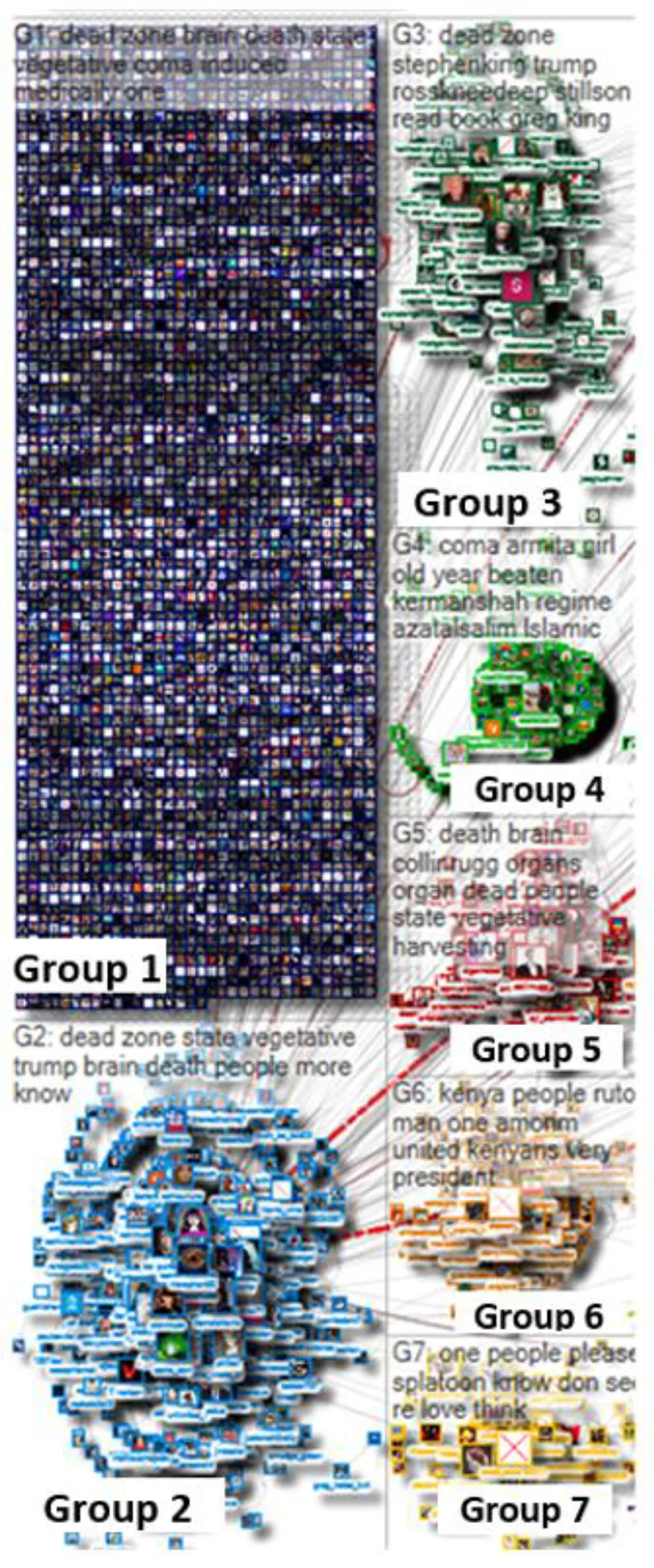
Social network structure of X posts retrieved using disorders-of-consciousness keywords, January 1 – December 31, 2023. Network visualization was produced in NodeXL Pro using the Harel-Koren Fast Multiscale layout algorithm with community detection by the Clauset-Newman-Moore modularity-optimization method. The seven largest communities (Groups 1–7) are shown in descending order of node count, with each group annotated with its most frequent terms. Node colors indicate group membership; edges represent reply, quote, and repost interactions (likes excluded). Group 1, the largest community, consists predominantly of isolates (users posting without engaging with others). Groups 2, 3, 6, and 7 are dominated by figurative, political, or popular-culture uses of disorders-of-consciousness terminology. Groups 1, 4, and 5 contain the substantive clinical, advocacy, and ethical content. See Results for detailed thematic interpretation.

Social network analysis identified seven principal user communities, presented in Figure 1 in descending order of size (Group 1 = largest).

#### Group 1:

Largest community; top terms: “dead zone,” “brain death,” “state,” “vegetative,” “coma,” “induced,” “medically.” This group represents an Isolates group ; predominantly composed of users posting in isolation (no reply/quote engagement) on a heterogeneous mix of clinical (“medically induced coma,” “vegetative state”) and figurative (“dead zone”) uses.

#### Group 2:

Top terms: “dead zone,” “state,” “vegetative,” “trump,” “brain,” “death,” “people,” “more,” “know.” Community Cluster / Polarized Crowd; political discourse intersecting with figurative use of DoC terminology, with references to political figures including Donald Trump.

#### Group 3:

Top terms: “dead zone,” “stephenking,” “trump,” “rosskneedeep,” “stillson,” “read,” “book,” “greg,” “king.” Brand/Topic Cluster centered on Stephen King’s novel *The Dead Zone*, with intersecting political content (Greg Stillson is a fictional populist politician in the novel, often invoked as an analog for current US political figures).

#### Group 4:

Top terms: “coma,” “armita,” “girl,” “old,” “year,” “beaten,” “kermanshah,” “regime,” “azatalsalim,” “lslamic.” Community Cluster representing an advocacy network coalescing around the human-rights case of Armita Garavand, a 16-year-old Iranian girl who entered coma after an alleged altercation with Iranian morality police in October 2023.

#### Group 5:

Top terms: “death,” “brain,” “collinrugg,” “organs,” “organ,” “dead,” “people,” “state,” “vegetative,” “harvesting.”Community Cluster centered on organ-donation/procurement discourse, with notable conspiratorial framing of “organ harvesting” intersecting with brain-death determination.

#### Group 6:

Top terms: “kenya,” “people,” “ruto,” “man,” “one,” “amorim,” “united,” “kenyans,” “very,” “president.” Community Cluster of national political discourse in Kenya in which DoC terminology was used figuratively.

#### Group 7:

Top terms: “one,” “people,” “please,” “splatoon,” “know,” “don,” “see,” “re,” “love,” “think.” Community Cluster around the Splatoon video game (“dead zone” gameplay mechanics); minimally relevant to clinical DoC discussion.

Across the seven communities, only Groups 1, 4, and 5 contained substantive clinical or clinical-adjacent DoC discourse; Groups 2, 3, 6, and 7 were dominated by figurative or non-medical uses of DoC terminology.

### Topic Structure

**Figure 2.**
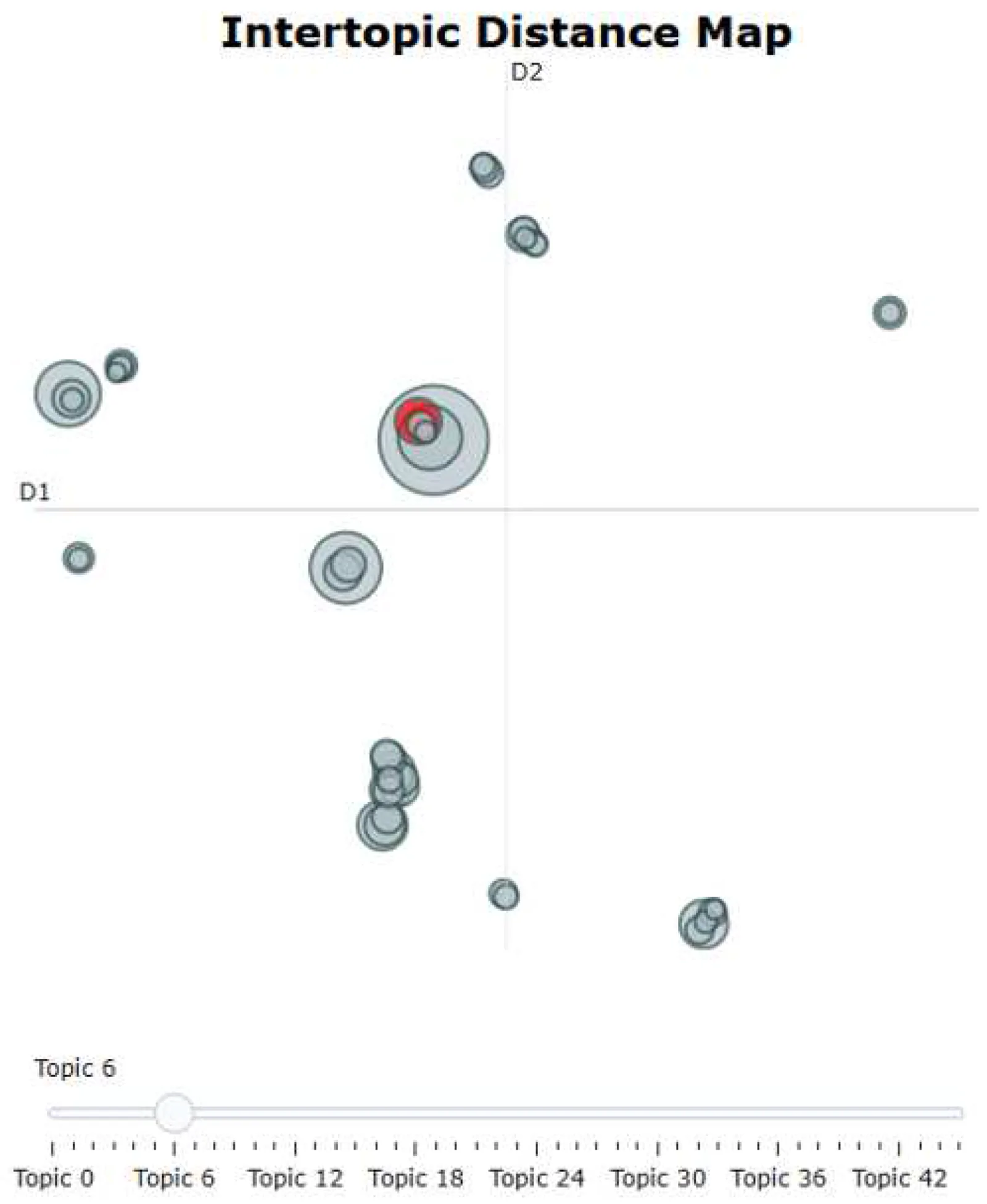
BERTopic intertopic distance map of disorders-of-consciousness-related X posts, 2023. Each circle represents one topic identified by the BERTopic transformer-based topic-modeling pipeline (sentence embedding via *all-MiniLM-L6-v2*, dimensionality reduction via UMAP, density-based clustering via HDBSCAN). Circles are projected into a two-dimensional UMAP space (axes D1, D2); inter-circle distances approximate semantic dissimilarity, with proximate topics sharing lexical and contextual content. Circle area is proportional to the number of posts assigned to each topic. The highlighted (red) topic (Topic 5) corresponds to the “dead zone — wifi / cell / phone service” non-medical figurative use. The clustering of multiple topics along the D1 axis reflects the dominant figurative-versus-clinical lexical gradient identified in the thematic synthesis. A complete topic-by-top-term table is provided in Supplementary Table S1.

BERTopic identified 50 coherent topics. Figure 2 presents the intertopic distance map (UMAP-projected first two dimensions, D1 × D2). A complete topic-by-top-term table with per-topic post counts is provided in Supplementary Table S1. Full topic numbering used below is preserved from the BERTopic output; topics not explicitly discussed are detailed in the Supplement.

### Thematic Synthesis

#### Theme 1 - Medical and Clinical Discourse.

A substantive portion of the corpus directly addressed the medical and clinical aspects of DoC. The *vegetative state* appeared as a focal concept across multiple topics (Topics 1, 3, 21, 29), with associated discussion of consciousness assessment, diagnosis, medical intervention, and legal implications. *Brain death* appeared as an additional clinical focus (Topics 4, 26, 27), with content centered on diagnostic criteria and downstream procedures. *Locked-in syndrome* appeared as a distinct cluster (Topic 14), demonstrating that the model could differentiate phenotypically related but mechanistically distinct conditions. *Medically induced coma* appeared in Topic 0, reflecting public awareness of this specific clinical practice. Specific named cases - most prominently Armita Garavand (Topic 7) -anchored otherwise abstract clinical concepts to identifiable individuals, a pattern previously described in social-media discourse on neurological conditions where high-profile individual cases drive transient surges in public engagement.^11^

#### Theme 2 - Ethical, Legal, and Societal Discourse.

A second substantial portion of the corpus engaged the ethical and legal dimensions of DoC. *Organ procurement and “organ harvesting”* emerged as a recurring topic (Topic 2) frequently coupled to brain-death discourse, reflecting both legitimate ethical debate and conspiratorial framing identified within Group 5 of the network analysis. The intersection of DoC with beginning-of-life and end-of-life debates was evident in Topic 21 (“fetus,” “abortion,” “baby life,” “vegetative state”). Topic 29 connected “rape,” “penalty,” “bill,” and “vegetative state,” reflecting public engagement with criminal-justice cases in which severe brain injury was a consequence of violent assault, a thematic cluster that, in 2023, was substantially anchored to high-profile legal proceedings in South Asia and the United States.

#### Theme 3 - Figurative and Non-Medical Use.

A third major component of the retrieved corpus consisted of non-medical uses of DoC-related terminology, dominated by the phrase “dead zone” across Topics 10, 11, 13, 16, 17, 25, 31, and 32. Specific non-medical contexts included connectivity dead zones (“wifi,” “phone service,” “cell” — Topic 5), gaming controller “stick drift”/dead-zone technical discourse (Topic 18), film and television uses (Topics 28, 32, anchored to the Stephen King novel/series *The Dead Zone*). By inspection, non-medical uses constituted the plurality of the figurative cluster and meaningfully diluted the corpus’s signal-to-noise ratio for clinical analysis.

### Cross-Cutting Observation - Algorithmic Inability to Disambiguate Context.

A clinically important secondary finding was that the general-purpose embedding model used by BERTopic (*all-MiniLM-L6-v2*) could not reliably separate posts that *substantively discussed* DoC from posts that merely *invoked* DoC terminology in passing, for example, anecdotal description of an unrelated individual as “in a coma” without further medical content. The short text length of X posts, the use of clinical terms in cultural shorthand, and the use of *all-MiniLM-L6-v2* (trained on general web text rather than biomedical corpora) all plausibly contributed. A domain-specialized biomedical encoder (e.g., PubMedBERT, BioBERT) would be an appropriate target for future replication.

## DISCUSSION

This study provides an English-language, single-platform comprehensive thematic analysis of the X social media landscape surrounding disorders of consciousness. Analysis of 30,621 posts from calendar year 2023 demonstrates that public discourse about DoC is substantial in volume, structurally heterogeneous, and extends well beyond purely clinical content. Three discourse domains dominated: medical/clinical, ethical/legal/societal, and figurative/non-medical. Each carries distinct implications for how DoC information is interpreted and potentially misunderstood by lay audiences.

A central interpretive distinction is between posts that substantively engage with DoC as a clinical, legal, or ethical entity and posts that merely *describe* an individual as being in such a state — or use the terminology metaphorically — without medical content. The latter category, while contributing to total content volume, does not constitute misinformation in itself; rather, it forms the ambient communication environment in which authoritative clinical content must compete for attention. This distinction is essential to accurately interpret the social-media misinformation landscape and to design effective public-facing interventions.

Within the medical/clinical theme, our findings reinforce the public salience of the *vegetative state* and *brain death* as focal points of public conversation. The international heterogeneity in coma definitions documented by the Curing Coma Campaign survey^8^ is mirrored in the variability of lay use of these terms, and the documented gaps in clinician- and family-facing educational resources ^10^are visible in the volume of clinically relevant content driven by named cases (e.g., Armita Garavand, Topic 7) rather than by systematically disseminated educational material.

Within the ethical/legal theme, the prominence of *organ harvesting* discourse adjacent to brain-death content — including in the conspiracy-leaning cluster of Group 5 is consistent with prior work in the Japanese-language YouTube/X ecosystem^11^and signals that public engagement with brain-death determination remains entangled with non-clinical concerns about organ procurement. The connection between *vegetative state* and discussions of criminal justice (rape; sentencing) Topic 29 represents a discourse pathway whose magnitude has not, to our knowledge, been previously quantified in English-language social media; this represents an actionable opportunity for clinician-advocate engagement when high-profile cases enter the public conversation.

Within the figurative/non-medical theme, the magnitude of “dead zone” content originating in popular culture and technology has methodological implications for any future Twitter/X-based DoC research. Investigators using broad lexical retrieval should anticipate that a substantial portion of any DoC corpus will be non-medical; either domain-specialized embedding models, downstream classifier-based filtering, or narrower keyword strategies will be required to address this.

Integrating across the SNA and topic-modeling results, the seven user communities mapped only loosely onto the topic-level clinical/non-clinical distinction: meaningfully clinical content was concentrated in Groups 1, 4, and 5, while Groups 2, 3, 6, and 7 were dominated by figurative or politically framed uses. This pattern suggests that public DoC discourse on X is not currently organized around clinically authoritative hubs and that intentional cultivation of such hubs by neurocritical care societies, patient organizations, and academic medical centers is a defensible communication-strategy goal.

Our findings build on a small but growing literature on social-media perceptions of DoC. Kondziella and colleagues’ crowdsourced lay-public survey^12^ identified marked confusion regarding awareness, prognosis, and the relationship between vegetative state and unresponsive wakefulness syndrome; our discourse-level findings are consistent with that survey’s documented lay-level conceptual confusion. Vargas Meza and Oikawa’s Japanese-language YouTube/X analysis^11^ documented high prevalence of non-informative or actively misinformative “brain death” content; our English-language X data reproduce similar patterns at scale, notably including the entanglement of brain-death discourse with conspiratorial “organ harvesting” framing.

### Limitations.

This study has several limitations that warrant emphasis. First, and most importantly, we did not adjudicate post-level factual accuracy against a clinical reference standard; we therefore report the discursive context within which DoC misinformation can circulate, not direct prevalence estimates of misinformation. Future work pairing the present landscape analysis with clinician-led accuracy coding of stratified random post samples would directly address this gap. Second, our retrieval was confined to one platform (X) and one language (English); generalization to Facebook, Reddit, TikTok, YouTube, or to non-English DoC discourse cannot be assumed. Third, retrieval was performed approximately 10 months after the end of the analytic window; posts subsequently deleted, withheld, or removed via content moderation may be undersampled. Fourth, the general-purpose *all-MiniLM-L6-v2* embedding model is not domain-specialized for biomedical text and likely contributed to the model’s inability to algorithmically distinguish clinical from figurative uses of overlapping terms; a domain-specific encoder (PubMedBERT, BioBERT, SapBERT) would be a logical replication target. Fifth, X’s API access was substantially restructured during 2023; the completeness of retrieval relative to the underlying population of matching posts depends on the API tier and rate-limit ceilings in effect at the time of extraction. Sixth, the original keyword glossary included “Dead Zone,” which produced substantial non-medical retrieval; we retained this term for transparency but flag that re-running with a narrower glossary would meaningfully reduce noise. Seventh, we did not apply automated bot/automation detection; results should be interpreted in light of possible inorganic activity. Finally, the structured thematic synthesis was performed by clinician-authors with subject-matter expertise but with a US-centric perspective; some non-US politically anchored clusters (e.g., Group 6, Kenyan political discourse) may benefit from regionally informed re-interpretation.

## CONCLUSIONS

Our analysis of more than 30,000 X posts published in 2023 demonstrates that public discourse about disorders of consciousness is substantial in volume, structurally heterogeneous, and embedded within emotionally charged, legally complex, and figuratively laden contexts. Three discourse domains dominate medical/clinical, ethical/legal/societal, and figurative/non-medical and unsupervised transformer-based topic modeling using a general-purpose embedding model cannot reliably separate substantive clinical content from incidental or metaphorical term-use. These findings provide an evidence-based map of the public communication environment in which DoC misinformation can propagate. Targeted neurocritical care educational, advocacy, and counter-misinformation efforts should be designed against this realistic landscape, with explicit attention to the high-engagement contexts (organ donation, criminal justice, named cases) within which clinical concepts are most likely to be encountered by lay audiences. Cross-platform, multilingual, and domain-encoder-based replication, together with direct accuracy coding, are the next logical steps.

## Supplementary Material

This is a list of supplementary files associated with this preprint. Click to download.

- STROBEChecklistDoCSocialMedia.docx
- STROBEChecklistDoCSocialMedia.docx
- SupplementaryTableS1.docx
- SupplementaryTableS1.docx
- SupplementaryTableS2CuringComaCollaborators.pdf
- SupplementaryTableS2CuringComaCollaborators.pdf

Supplementary Table S1. Complete BERTopic topic-by-top-term table with per-topic post counts.

Supplementary Table S2. Curing Coma Campaign Community of Collaborators member list.

Supplementary File. Completed STROBE reporting checklist.

## Figures and Tables

**Figure 3.**
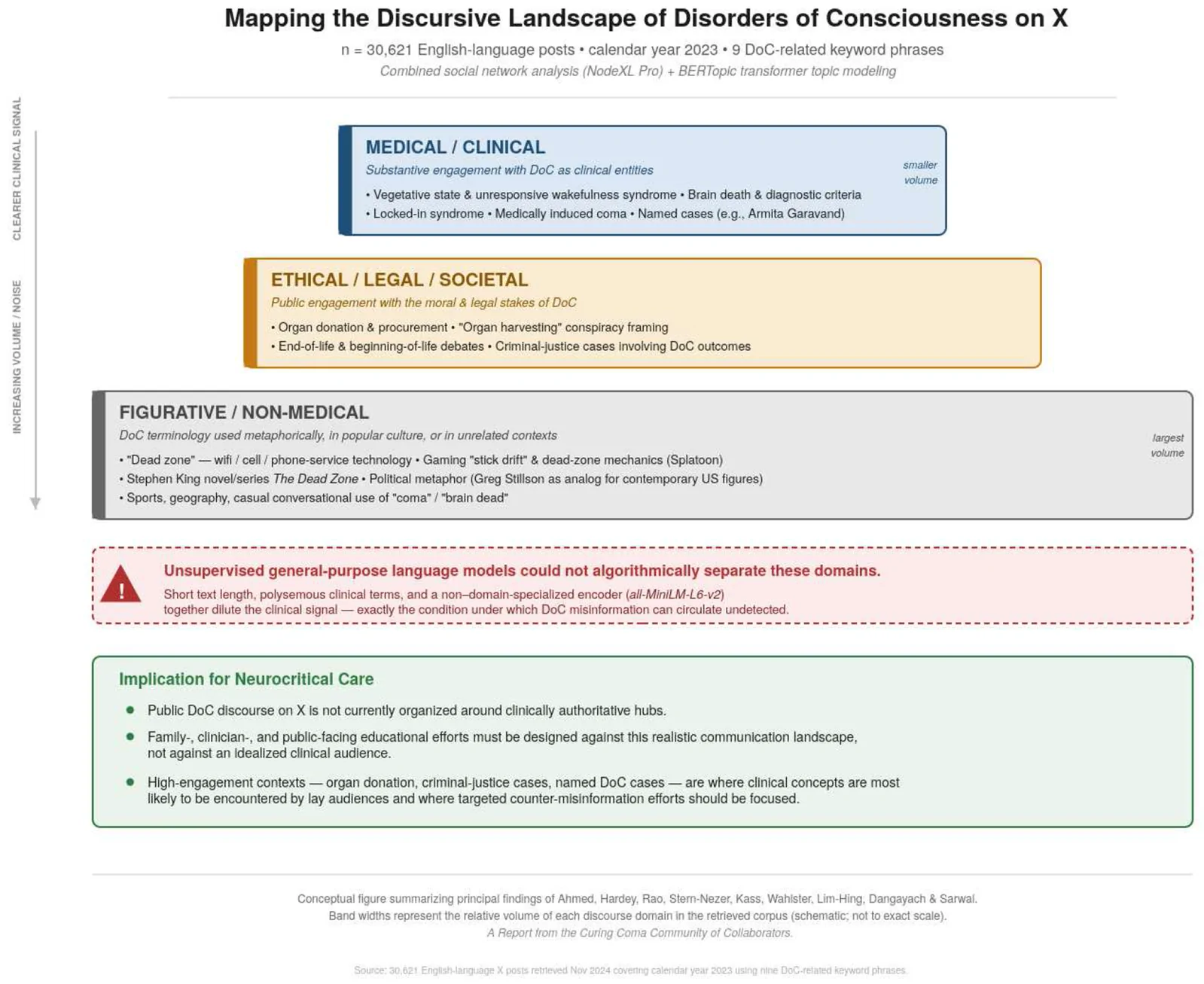
Conceptual figure summarizing principal findings. Bandwidth represent the relative volumes of each discourse domain in the retrieved corpus (schematic, not to exact scale)

## Data Availability

In accordance with the X Developer Agreement and Policy, individual post content cannot be redistributed; post identifiers (status IDs) and analysis code will be made available from the corresponding author upon reasonable request, subject to platform terms of service in effect at the time of any such request.
